# Long-term outcomes after combined arthroscopic medial reefing and lateral release in patients with recurrent patellar instability – a retrospective analysis

**DOI:** 10.1186/s12891-017-1636-8

**Published:** 2017-06-24

**Authors:** Dominik Schorn, Sera Yang-Strathoff, Georg Gosheger, Tim Vogler, Sebastian Klingebiel, Carolin Rickert, Dimosthenis Andreou, Dennis Liem

**Affiliations:** Department of General Orthopedics and Tumor Orthopedics, Münster University Hospital, Albert-Schweitzer-Campus 1, 48149 Münster, Germany

**Keywords:** Recurrent patellofemoral instability, Lateral release, Medial reefing, Risk factors, Functional outcome, Residual complaints

## Abstract

**Background:**

There is currently no consensus regarding the optimal surgical treatment method for patients with recurrent patella instability. Our goal was to evaluate the long-term results of combined arthroscopic medial reefing and lateral release, to identify possible risk factors for recurrent dislocations and residual complaints after surgical treatment and to assess functional outcome.

**Methods:**

We performed a retrospective study of 38 patients (43 knees) treated with all-inside technique between 2001 and 2010. The functional outcome was evaluated with the Kujala score, while pain intensity was scored on a visual analogue scale (VAS). Contingency tables were analysed with Fisher’s exact test. Non-parametric analyses were carried out with the Mann-Whitney U and the Wilcoxon signed-rank test. Survival curves were calculated with the Kaplan-Meier method and compared with the log-rank test.

**Results:**

The median age at surgery was 16 years (range, 9–44 years) and the median follow-up amounted to 9.7 years (range, 4.7–14.7 years). Residual complaints were present in 34 cases (79%). Patients with residual complaints had a trend for a higher body mass index (BMI) at surgery (25.7 vs. 21.6, *P* = .086). Twenty-two cases had recurrent dislocation after a median interval of 30 months. The probability of recurrent dislocations amounted to 16% after 1 year and 52% after 10 years. There were no significant differences in the presence of residual complaints (*P* = .721) and median VAS score (*P* = .313) between patients with or without recurrent dislocation. Patients with recurrent dislocations had a trend towards younger age at surgery (15 vs. 18 years, *P* = .076). The median Kujala score of the affected knee was 81. Patients with recurrent dislocations had a significantly lower score compared to patients without recurrent dislocations (67 vs. 91, *P* < .001).

**Conclusions:**

The combined arthroscopic lateral release with medial reefing does not appear to be an adequate treatment for patients with chronic patellar instability in long-term follow-up. Younger patients might be at a higher risk for recurrent dislocations, while a higher BMI at surgery might be associated with residual complaints.

## Background

Chronic patellofemoral instability is a complex problem influenced by a number of factors, such as limb alignment, the congruency between patella and trochlea and the sufficiency of the surrounding soft tissue structures [1, 2]. Following an acute dislocation, which often leads to a rupture of the medial stabilizers, 15-44% of the patients will develop a recurrence with conservative treatment [3]. Patients with a second dislocation have a 50% probability to develop chronic patellofemoral instability [1], with an increasing likelihood for chondral lesions and the development of secondary arthritis [4, 5]. Surgical treatment is therefore recommended for patients with recurrent dislocations [2].

The first surgical soft tissue procedure addressing patellofemoral instability was described at the end of the nineteenth century as a variation of medial reefing [6]. Since then, more than 100 different surgical techniques have been described [2], and there is currently no consensus regarding the optimal surgical treatment method for patients with chronic instability in the literature [1]. Variations of soft tissue procedures including a lateral release and/or medial reefing were commonly used in the absence of bony deformity over the last three decades to achieve proximal realignment [2, 7–10], while newer reconstructive procedures based on tendon transfers for reconstruction of the medial patellofemoral ligament (MPFL) have gained in importance in recent years [2, 4, 11, 12].

Several studies have demonstrated that an isolated lateral release is associated with high rates of recurrent dislocations and poor clinical outcomes [1, 2, 8]. On the other hand, the combination of lateral release with medial soft tissue reefing, appeared to lead to a decrease in recurrent instability rates and an increase of patient satisfaction [9]. Several authors have reported on good short- to mid-term results with all-arthroscopic or arthroscopically assisted minimal invasive procedures [13–17]. However, no studies have evaluated, to our knowledge, the long-term results of a combined arthroscopic lateral release with medial reefing, which was the main objective of our study. Secondary objectives were the identification of possible risk factors for recurrent dislocations and residual complaints after surgical treatment, as well the assessment of functional outcome. We hypothesized that the failure rate might increase over time, and that patient age, body mass index (BMI), as well as previous surgical procedures would affect the probability of failure and functional results.

## Methods

### Study design

We performed a single center retrospective analysis of patients with chronic patella instability treated with an arthroscopic medial reefing combined with a lateral release. The inclusion criteria were occurrence of recurrent lateral patellar dislocation, a tibial tuberosity trochlear groove (TTTG) distance under 20 mm and a knee valgus angle of <10 degrees. Exclusion criteria were the presence of a Dejour type c or type d trochlear dysplasia, severe neuromuscular or congenital diseases.

Between January 2001 and July 2010 51 consecutive patients with chronic patella instability meeting these criteria that were surgically treated at our institution were identified through our surgical database. All of these patients were contacted by telephone between March and May 2015 and asked to take part in the study. 38 (73%) patients agreed to participate.

Participating patients received a link for a specially designed online questionnaire (www.umfrageonline.com, enuvo GmbH, Zurich, Switzerland) per email and made a telephone appointment with one of the study investigators. In order to minimize the risk of misinterpretations, the questionnaire was filled out online during this second telephone call, in order for the patients to be able to clarify any issues that might arise. Data regarding patient demographics, disease-specific patient history, further dislocations following surgery and treatment thereof, as well as present pain, scored on a visual analogue scale (VAS), were documented.

The functional outcome was evaluated with the Kujala Anterior Knee Pain Scale (AKPS) [18]. The AKPS is a recognized and established self-report questionnaire to evaluate patients with disorders of the knee, especially disorders of the patellofemoral joint. It consists of 13 items documenting response to six activities potentially causing knee complaints (walking, running, jumping, climbing stairs, squatting, sitting with bended knees) and associated symptoms such as limitation of range of motion, limp and swelling. The maximum achievable score is 100. Lower scores indicate stronger complaints and limitations [19]. Its test-retest reliability and validity is proven to be good by Kujala et al. and Timm et al. [18, 20] A score < 64 is considered to be a poor result, 65 to 84 a fair, 85 to 94 a good and >94 an excellent result [21].

### Surgical technique

**Fig. 1:**
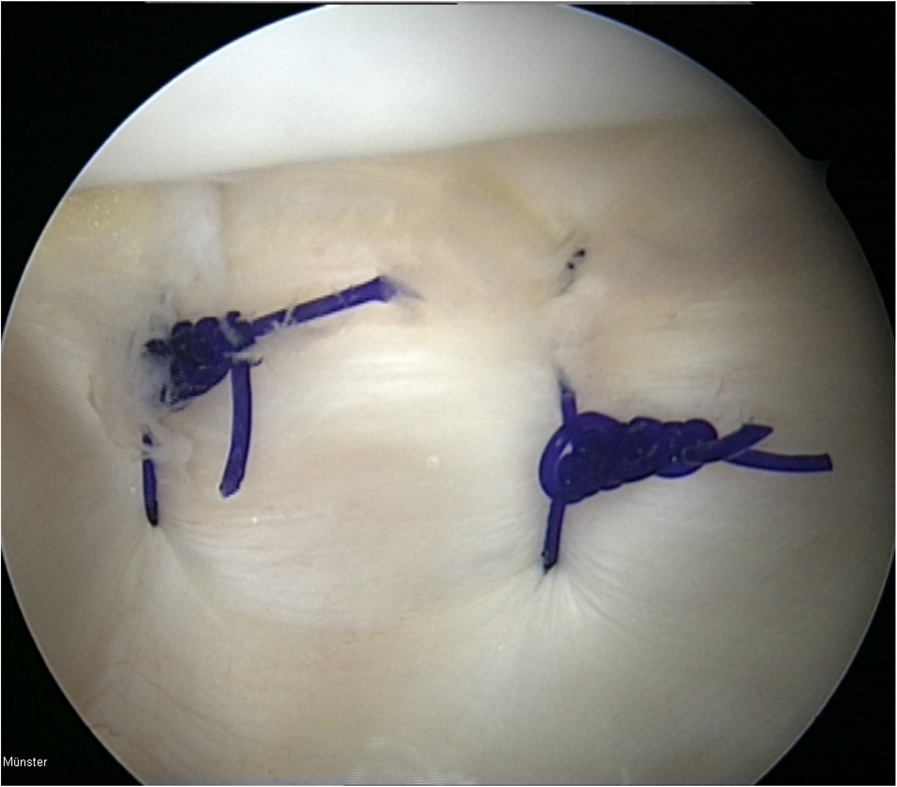
Arthroscopic view of PDS suture loops for medial reefing

The patients were surgically treated by four consultant surgeons. In all patients a combined medial reefing and lateral release was performed by the technique first published by Jeffrey Halbrecht [14], an entirely arthroscopic all-inside procedure. The surgery was done under general anaesthesia. A tourniquet was inflated and the leg was placed in a thigh holder. Following a diagnostic arthroscopy using a standard anterolateral portal, an anteromedial portal was established and the medial retinaculum was gently trimmed to induce healing response using a shaver system. An additional superolateral portal with cannula was established for suture management. The sutures for the medial reefing were inserted percutaneously with a hollow epidural needle, which was placed to the medial edge of the patella parallel to its chondral surface. A No. 0 polydioxanone suture (PDS) was passed through the needle and retrieved through the superolateral portal. The needle was retracted subcutaneously and then reinserted through the medial retinaculum 2 cm posteriorly in a more vertical angle. A suture loop was created by retrieving the second end of the suture through the superolateral portal. This process was repeated 3 to 5 times, depending on the size of the patella (Fig. 1). Subsequently a release of the lateral retinaculum was performed 5 to 10 mm from the lateral edge of the patella by using an electrosurgical cutting and coagulation device. Finally, the sutures were tied arthroscopically inside the joint.

During inpatient treatment patients received physical therapy daily and performed active assisted exercises, while regular physical therapy sessions at least twice per week were recommended afterwards. Knee mobilization was restricted by a brace to 30°, 60° and 90° flexion for 2 weeks each and patients were allowed partial weight bearing of 20 kg for 6 weeks. Subsequently intensive strengthening exercises primarily with eccentric training and proprioceptive exercises were recommended.

### Statistical analysis

The duration of follow-up and the time to recurrent dislocation were calculated from the date of surgical treatment. Contingency tables were analysed with Fisher’s exact test. Non-parametric analyses were carried out with the Mann-Whitney U and the Wilcoxon signed-rank test. Survival curves were calculated with the Kaplan-Meier method and compared with the log-rank test [22, 23]. Statistical analyses were performed with the IBM SPSS Statistics software version 21.0 (IBM Corp., Armonk, NY). All *P* values are two-sided; a *P* value < .05 was considered significant.

## Results

There were 29 female and 9 male patients. 5 patients had been operated on both knees, leading to 43 cases as the subject of this analysis (Table 1). The median age at the time of surgery was 16 years (range, 9–44 years). The median patient body mass index at surgery was 25.2 kg/m^2^ (range, 17.8–41.5 kg/m^2^). The affected knee had undergone previous surgery in 8 cases. The median follow-up amounted to 9.7 years (range, 4.7–14.7 years).

**Table 1:** participant characteristics and sex related differences

	All cases (*n* = 43)	Men (*n* = 11)	Women (*n* = 32)	*P* value
Age (y)	16 (9-44)	16 (14-28)	16 (9-44)	.264
BMI (kg/m^2^)	25.2 (17.8-41.5)	27.1 (25.3-38.0)	23.9 (17.8-41.5)	.019
Previous surgery of the affected knee	8	1	7	.656
Residual complaints	34	8	26	.672
Recurrent dislocations	22	7	15	.488
Kujala score	81 (39-100)	88 (62-100)	76 (39-100)	.042

**Fig. 2:**
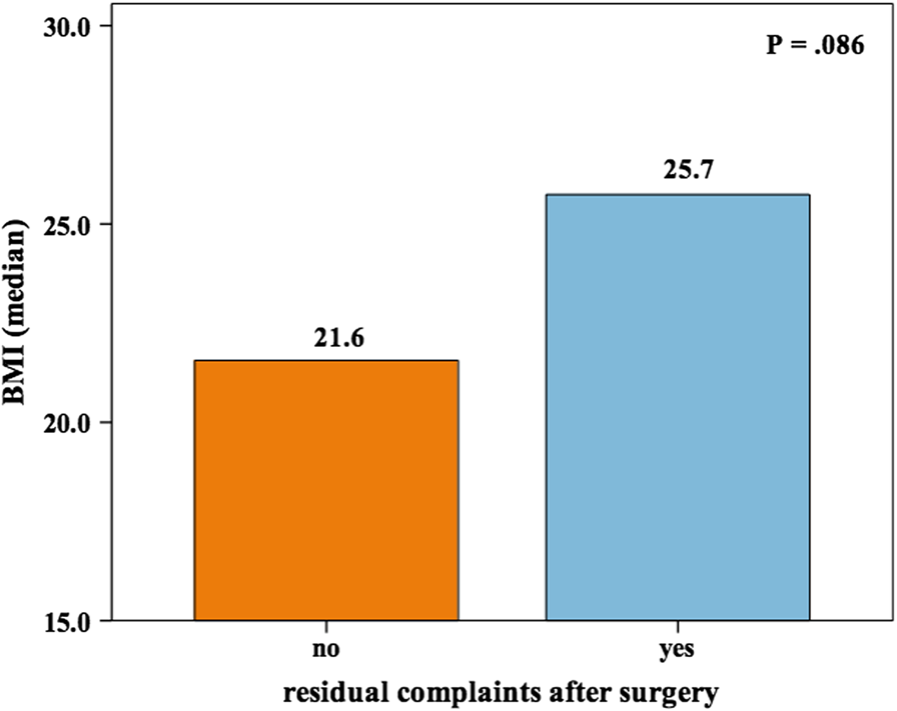
Residual complaints after surgery according to patients` BMI

At the time of the survey, residual complaints, such as pain or restricted range of motion in the affected knee were present in 34 cases. There was a trend for patients with residual complaints to have a higher BMI at the time of surgery, compared to patients with no residual complaints, which, with the number of patients available for this trial, did not reach statistical significance (25.7 vs. 21.6, *P* = .086 – Fig. 2). The median VAS score was 3 (range, 0–8) for cases with residual complaints. Three patients required pain medication regularly.

**Fig. 3:**
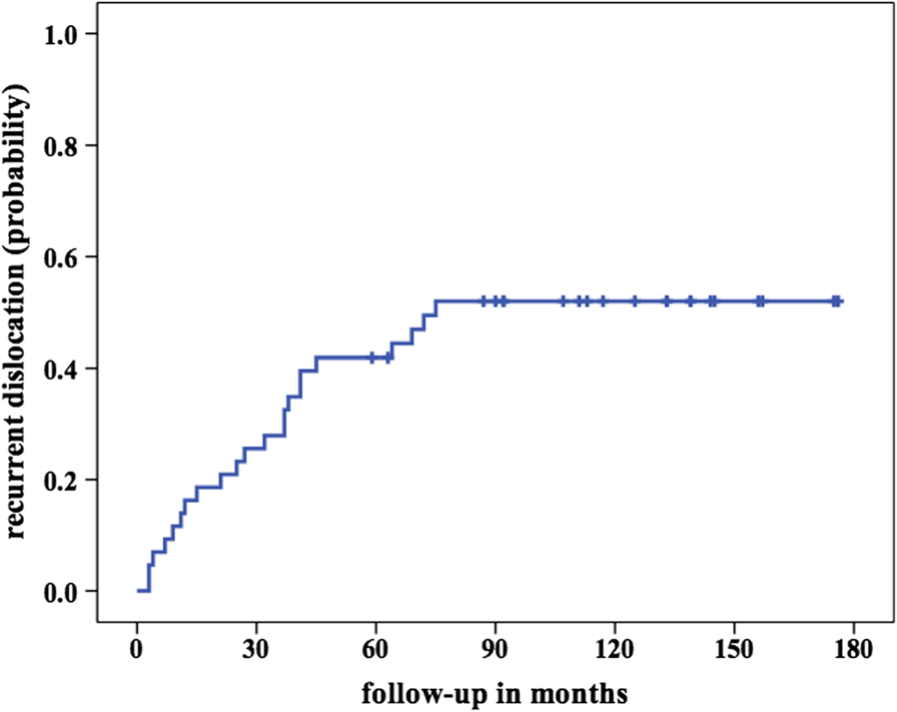
Probability of recurrent dislocation after surgery over time (16% after 1 year, 21% after 2 years, 28% after 3 years, 42% after 4 and 5 years, 52% after 10 years)

Twenty-two cases had a recurrent dislocation of the patella after a median interval of 30 months (range, 3–75 months), accounting for a recurrence rate of 51%. Only 4 of these dislocations were attributed to an acute traumatic event. The probability of recurrent patella dislocation following surgical treatment over time amounted to 16% after 1 year, 42% after 5 years and 52% after 10 years (Fig. 3). There were no significant differences in the presence of residual complaints (*P* = .721) and median VAS score (*P* = .313) between patients who developed recurrent dislocation and patients who did not. Further surgical procedures were necessary in 11 cases. A knee brace was regularly used in 11 cases, 3 of which had undergone further surgical procedures.

**Fig. 4:**
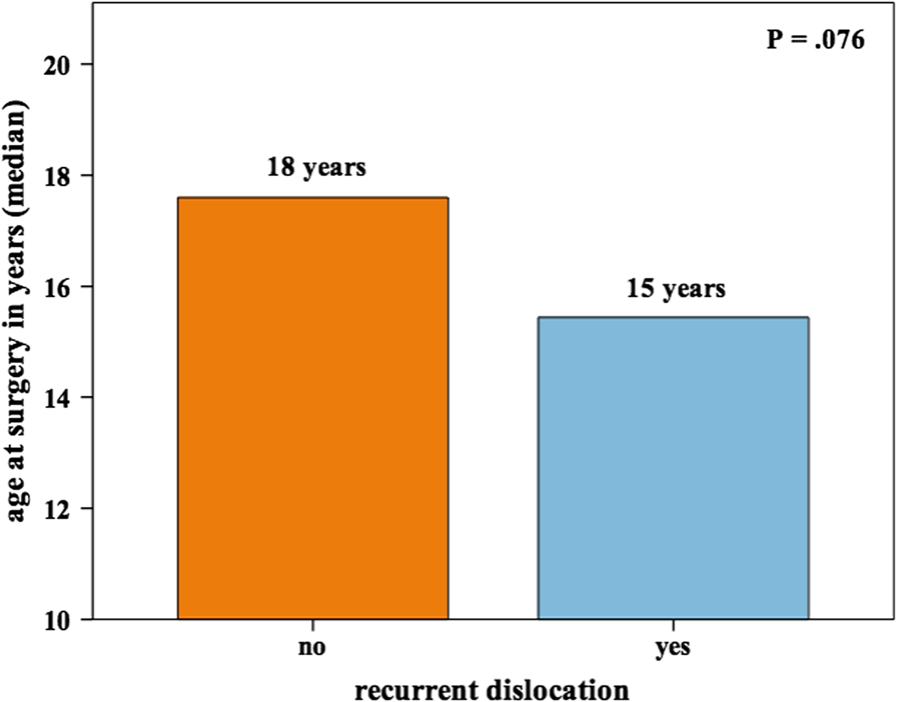
Recurrent dislocation after surgery according to patients` age

Regarding possible risk factors for recurrent dislocation, there were no significant differences in the probability of further dislocation between cases, which had had previous surgical treatment of the affected knee, and cases, which had not (14% and 46% vs. 25% and 37% after 1 and 5 years, *P* = .752). Patients with recurrent dislocations had a trend for a younger age at surgery (15 vs. 18 years, *P* = .076 – Fig. 4). There were no significant differences in the BMI of patients with or without recurrent dislocations (25.2 vs. 25.1, *P* = .679).

**Fig. 5:**
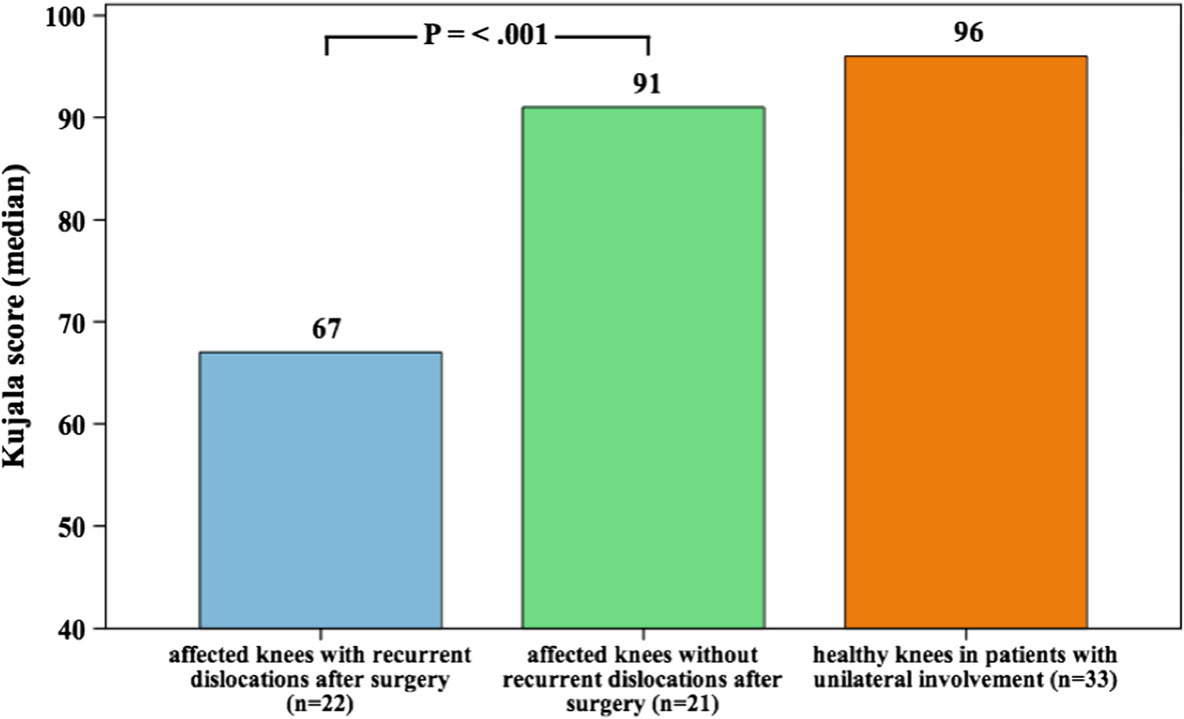
Median Kujala score according to knee status

The median Kujala AKPS score of the affected knee at the time of the survey amounted to 81 (range, 39 – 100). Female patients had a significantly lower score compared to male patients (Table 1). 5 cases had an excellent result, 13 cases a good, 15 cases a fair and 10 cases a poor result. Patients with recurrent dislocations had a significantly lower AKPS score compared to patients without recurrent dislocations (67 vs. 91, *P* < .001 – Fig. 5). Finally, the median AKPS score in the 33 patients with only one affected knee was significantly lower than the median AKPS score of the healthy knee (81 vs. 96, *P* < .001).

## Discussion

Various surgical procedures have been proposed for the treatment of patellofemoral instability [1, 2, 9]. However, the efficacy of most of these procedures remains a subject to debate and no technique has been established as the gold-standard treatment for patients with recurrent patella dislocations [1]. One of the well described and in the recent past commonly used surgical procedures is arthroscopic medial reefing combined with lateral release [1, 2, 9]. As no studies, to our knowledge, have evaluated the long-term outcome of this technique, we performed this analysis to assess the clinical outcome and the risk of recurrent dislocations after treatment, as well as to identify possible prognostic factors. Inevitably, the evolution of the surgical management of patellofemoral instability at our department and our strict inclusion criteria resulted in the relatively limited sample size of our analysis, which is one of the main limitations of this study. Further limitations were the study’s retrospective nature and the fact that 13 of the 51 patients who were surgically treated at our institution declined to participate to the study. On the other hand the same strict inclusion criteria provided us with a highly homogeneous patient cohort and limited the possible impact of confounding factors on our results.

One of the most interesting results of our study was the very high probability of recurrent dislocation, amounting to 42% after 5 years and 52% after 10 years. At first, this finding seems to contradict the results of previous studies, which demonstrated a significantly lower failure rate. Schottle et al. described a failure rate of 4%, while Small et al. a rate of 7% [15, 16]. However, the mean follow-up amounted to 12 months in the first study and 19 months in the second. On the other hand Zhao et al. reported on a failure rate of 26% after an average follow-up of 60 months, suggesting that the failure rates increase over time [11]. As far as we are aware the median follow-up of 9.7 years in our study is by far the longest reported in the literature, which might account for the higher failure rate in our patient cohort.

Following a first episode of patella subluxation or dislocation, patients experiencing a further episode of patella dislocation have a 50% probability to develop chronic patellofemoral instability [1, 24]. This high risk justifies the recommendation for an invasive surgical procedure, however, the results of our study suggest that arthroscopic medial reefing combined with lateral release is an insufficient approach with regards to long-term follow-up, as it appears to provide no superior results compared to conservative treatment in terms of joint stability [25].

One possible reason for the high failure rates in our study might be the lateral release aspect of the combined surgical procedure. Biomechanical studies on cadaveric knees have demonstrated that a lateral release has a negative effect on both medial and lateral stability [26, 27], and several studies have shown that an isolated lateral release yields poor results in terms of recurrence of instability, local complaints and patient satisfaction [1, 2, 8]. It is therefore questionable whether the combined surgical procedure offers any advantages to an isolated medial reefing. In a series of 25 cases with patellofemoral instability treated only with arthroscopically assisted medial reefing Miller et al. found no recurrent dislocations or subluxations and reported on a very high patients satisfaction after a mean follow-up of 5 years [28]. A further study by Boddula et al. evaluated the long-term results of medial reefing without lateral release in a series of 20 cases and an average follow-up of 11.8 years and demonstrated good functional outcomes with only one recurrent subluxation and no recurrent dislocations [7].

Another technique that has been proposed for the treatment of recurrent patellar dislocation is medial patellofemoral ligament reconstruction, which appears to provide superior results in terms of recurrent dislocation rate and functional outcome, compared to arthroscopic medial reefing combined with lateral release. Steiner et al. analysed a series of 34 patients with chronic patellar instability and trochlear dysplasia and found no recurrent dislocations after a mean follow-up of 67 months and a mean Kujala score of 91 [29]. Nomura et al. reported on the outcome of medial patellofemoral ligament reconstruction in a series of 24 cases with recurrent patellar dislocation after a mean follow-up of 11.9 years and found 2 cases with recurrent dislocations and a mean Kujala score of 94 [30].

Regarding possible risk factors for recurrent dislocations in our study, patients with failure of stabilization had a trend for a younger age at the time of surgery. Younger age has previously been identified as a risk factor for recurrent dislocations following primary patella dislocation. Balcarek et al. analysed a series of 61 patients treated conservatively following primary patella dislocation and found that patients experiencing recurrent dislocations in follow-up had a significantly younger age compared to patients who experienced no further dislocations, with 15 years at the time of diagnosis compared to 22 years [31]. A further study by Buchner et al. evaluated 126 patients undergoing conservative or surgical treatment for acute traumatic primary patella dislocation and showed that patients younger than 15 years had a significantly higher rate of recurrent dislocations compared to older patients, with 52% compared to 26% [32].

With regard to clinical outcome, there was a trend for patients with residual complaints at last follow-up to have a higher BMI at the time of surgery, compared to patients with no residual complaints. While no studies on the arthroscopic treatment of patellofemoral instability have evaluated, as far as we are aware, the impact of BMI at the time of surgery, several researchers have reported on an association between BMI and patient outcome following knee surgery. Erdil et al. evaluated the outcome of 1090 patients who underwent arthroscopic partial meniscectomy and found that overweight and obese patients with a BMI >26 had worse functional outcomes compared to non-obese patients [33]. Another study by Enderlein et al. reported on the results of a prospective series of 224 patients with recurrent patella dislocations undergoing MPFL reconstruction and found that patients with a BMI >30 had a worse functional outcome compared to patients with a lower BMI [34].

## Conclusion

In conclusion, the combined arthroscopic lateral release with medial reefing does not appear to be an adequate treatment of chronic patellofemoral instability in long-term follow-up. Younger patients might be at a higher risk for recurrent dislocations, while a higher BMI at the time of surgery might be associated with residual complaints. Taking into consideration the negative impact on patella stability and the poor clinical results of isolated lateral release, we believe that the combined arthroscopic procedure should no longer have a place in the management of patients with chronic patellofemoral instability.

## Abbreviations

AKPS: Anterior Knee Pain Scale
BMI: Body Mass Index
MPFL: Medial Patellofemoral Ligament
PDS: Polydioxanone Suture
TTTG: Tibial Tuberosity Trochlear Groove
VAS: Visual Analogue Scale
